# Genome Editing of Plant Mitochondrial and Chloroplast Genomes

**DOI:** 10.1093/pcp/pcad162

**Published:** 2023-12-19

**Authors:** Shin-ichi Arimura, Issei Nakazato

**Affiliations:** Laboratory of Plant Molecular Genetics, Graduate School of Agricultural and Life Science, The University of Tokyo, 1-1-1, Yayoi, Bunkyo-ku, Tokyo, 113-8657 Japan; Laboratory of Plant Molecular Genetics, Graduate School of Agricultural and Life Science, The University of Tokyo, 1-1-1, Yayoi, Bunkyo-ku, Tokyo, 113-8657 Japan

**Keywords:** Base editors, Chloroplasts, Genome editing, Mitochondria, Plastids, TALENs

## Abstract

Plastids (including chloroplasts) and mitochondria are remnants of endosymbiotic bacteria, yet they maintain their own genomes, which encode vital components for photosynthesis and respiration, respectively. Organellar genomes have distinctive features, such as being present as multicopies, being mostly inherited maternally, having characteristic genomic structures and undergoing frequent homologous recombination. To date, it has proven to be challenging to modify these genomes. For example, while CRISPR/Cas9 is a widely used system for editing nuclear genes, it has not yet been successfully applied to organellar genomes. Recently, however, precise gene-editing technologies have been successfully applied to organellar genomes. Protein-based enzymes, especially transcription activator–like effector nucleases (TALENs) and artificial enzymes utilizing DNA-binding domains of TALENs (TALEs), have been successfully used to modify these genomes by harnessing organellar-targeting signals. This short review introduces and discusses the use of targeted nucleases and base editors in organellar genomes, their effects and their potential applications in plant science and breeding.

## Introduction

**Fig. 1:**
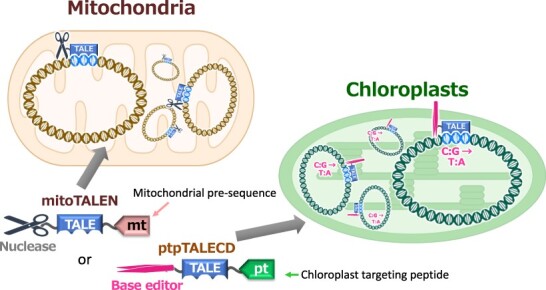
Strategies of organellar genome editing by protein-based genome-editing enzymes. An artificial targeted endonuclease, TALEN, with a mitochondrial presequence (mitoTALEN), is translated in the cytosol and imported into mitochondria to cut the target sequences in all copies of the multi-copious mitochondrial genomes. Base editing enzyme with a plastid-targeting peptide is transported into plastids to modify targeted cytosine to thymine.

The sizes of plastid (chloroplast) and mitochondrial genomes in land plants are typically hundreds of kb and encode less than 200 genes in total, which is less than 1% of the total number of genes of a plant. Plastid genomes encode some central components of photosynthesis (Allen 2015), including, e.g. the catalytic subunit of ribulose 1,5-bisphosphate carboxylase/oxygenase (Rubisco), an enzyme that rate limits photosynthetic carbon fixation (Gionfriddo et al. 2023). The mitochondrial genomes encode some central components of respiration as well as genes responsible for an agriculturally important trait exploited to produce F_1_ hybrid seeds, called cytoplasmic male sterility (CMS) (Hanson and Bentolila 2004, Allen 2015). To achieve a better understanding of and improvement in photosynthesis, respiration and CMS, it is thus essential to develop ways to modify organellar genomes (henceforth specifically referring to plastid and mitochondrial genomes). CRISPR/Cas9 and its related systems, which are commonly used to modify nuclear genomes, have not yet worked well in organellar genomes because of the difficulty of efficiently transporting the required guide RNAs into organelles (Son and Park 2022). However, proteins can be easily and efficiently delivered into organelles by just attaching the transporting signal sequences to their N-termini (Köhler et al. 1997, Lao et al. 1999) (**Fig. 1**). This review introduces the enzymes used to modify plant organellar genomes and describes some of their benefits.

## CRISPR-Free, Protein-Based Systems for Organellar Genome Editing

**Fig. 2:**
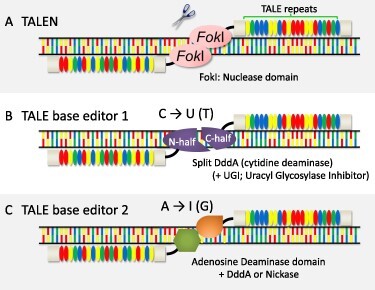
TALEN-based genome editing enzymes available for plant organellar genomes. (A) TALEN, the scissor enzyme. Dimerized *Fok*I domains cause the DSB of DNA in the sequence (target window) between the two sequences recognized by TALE repeat domains. (B) TALECD/DdCBE, a C-to-U (T) base editor. Paired molecules recognize the sequences up- and downstream of the target window, which includes a target base. A cytidine deaminase domain, DddA, is cut into two molecules, and N- and C-terminal halves are attached to the left and right TALE repeat domains, respectively. (C). mitoBE/TALED, A-to-G base editors. One of the two molecules has an adenosine deaminase domain, and the other has a domain that helps the activity of the adenosine deaminase. The ‘help domain’ is an (activity-weaken) DddA domain or a Nickase to open up the double-stranded DNA to single-stranded DNA.

A protein-only programmable target-specific editing system has two essential domains: a DNA-binding domain and a nuclease domain. Three major programmable target-specific nucleases that have distinct types of DNA-binding domains are available for editing mammalian mitochondrial DNA: transcription activator–like effector nucleases (TALENs)—the most frequently used of the three nucleases (Bacman et al. 2013), zinc finger nucleases (Minczuk et al. 2008) and meganucleases/homing endonucleases (I-CreI, Arcus) (Zekonyte et al. 2021). **Fig. 2A** shows the interaction between the target DNA sequence and TALENs. Each TALE repeat (33–35 amino acids) within the DNA-binding domain of TALENs recognizes its target nucleotide. TALENs have been more frequently used than the other two partly because assembling an array of TALE repeats to match the target sequence is relatively easy (Cermak et al. 2011, Sakuma et al. 2013) (kits for assembling TALENs can be obtained from Addgene, https://www.addgene.org/talen/), although it is more laborious and difficult than constructing a vector encoding the CRISPR/Cas9 enzyme. The TALE domain is derived from an effector of a plant-pathogenic bacterium in the genus *Xanthomonas*. The bacterium injects the effector into the cytosol of host plant cells, after which the effector acts as a transcription factor to express sugar transporters that exude sucrose outside of the cells (Zhang et al. 2022). A TALEN is usually composed of two protein molecules (**Fig. 2A**). The target between the sequences recognized by the TALE domains is cut by dimerized FokI nuclease domains, resulting in a DNA double-strand break (DSB). In the case of editing organellar DNAs, a mitochondrial presequence or a plastid-targeting signal is attached to the N-terminus of the proteins to deliver an artificial enzyme into the mitochondria or plastids (Bacman et al. 2013, Huang et al. 2021). In many cases, DNA or mRNA molecules encoding organellar-targeting TALENs are introduced into the cells instead.

Recently, another gene-editing enzyme called a base editor, which changes C:G pairs to T:A pairs or changes T:A pairs to C:G pairs, has been applied to modify organellar genomes in animals and plants (Mok et al. 2020, Kang et al. 2021, Nakazato et al. 2021, 2022, Cho et al. 2022). Targeted single-base editing causes the smallest change in the genome, which would be a good choice for the precise analysis of organellar genomes and crop breeding. Such single-nucleotide polymorphisms (SNPs) that have beneficial effects on plants can then be transferred to other plants and crops through crop breeding. Base editing can also generate amino acid substitutions to modify the function of proteins or create a premature stop codon to knock out a gene. It can also modify the three-dimensional structure of a DNA or RNA and prevent *trans*-factor proteins from binding to their target *cis*-sequences. With TALE-based base editors that target organellar genomes, a cytidine (or adenine) deaminase domain is attached to the TALE domain instead of the Fok I nuclease domain (**Fig. 2B and C**). Similarly, the CRISPR/Cas-based base editors targeting the nucleus also have cytidine or adenine deaminase domains (Komor et al. 2016, Nishida et al. 2016, Gaudelli et al. 2017), but they have different substrate preferences. Since the CRISPR/Cas ribonucleoprotein unwinds double-stranded DNA (dsDNA), the cytidine deaminases used in CRISPR/Cas-based base editors use single-stranded DNA (ssDNA) as a substrate. On the other hand, the TALE domain recognizes its target sequence by surrounding the major groove of the dsDNA without unwinding it, and therefore, the cytidine deaminase domains used in organelle-targeting TALE-based base editors use dsDNA as a substrate. Mok et al. (2020) identified the cytidine deaminase domain operating on dsDNA, named DddA, from a bacterial toxin from *Burkholderia cenocepacia*. They split DddA into two pieces and fused them to a TALE domain and a uracil glycosylase inhibitor to make a DddA-derived cytosine base editor (DdCBE, **Fig. 2B**). Using a pair of DdCBEs that have base editing activity only in the target window, they succeeded in converting target cytosines to thymines in the mitochondrial DNA of human cells. While DdCBE with DddA tends to convert C on the 3ʹ side of T (TC) in the animal mitochondrial genome (Mok et al. 2020, Guo et al. 2021, Lee et al. 2021), modified versions of DdCBE, which have an in vitro–evolved DddA or a paralog of DddA isolated from another bacterium, can use ac, GC and CC as substrates in the mammalian mitochondrial genome (Mok et al. 2022a, Guo et al. 2023, Mi et al. 2023). DdCBE has been demonstrated to effectively edit target cytosines in plant organellar genomes, as evidenced by research conducted by Kang et al. (2021), Li et al. (2021), and Nakazato et al. (2021), (2022), (2023). More recently, Hu et al. (2023) successfully achieved strand-specific C-to-T base editing in both the nuclear and plastid genomes of rice, as well as in the human mitochondrial genome, utilizing a base editor known as CyDENT.

Adenosine is converted to inosine in the first step of A-to-G base editing. The inosine is paired with cytosine during DNA replication, resulting in a G:C pair. Although an adenosine deaminase targeting dsDNA has not yet been identified, A:T to G:C substitution in the mitochondrial genome of human cells was achieved by combining TadA, an adenine deaminase targeting ssDNA, and a TALE domain with DddA [in the case of TALE-linked deaminases (TALEDs)] or a nickase [in the case of mitochondrial DNA base editors (mitoBEs)] like in **Fig. 2C** (Cho et al. 2022, Yi et al. 2023). In such cases, TadA could access ssDNA, possibly because DddA with reduced activity unwound dsDNA or because a nickase cut only one strand of the dsDNA. We noted that DddA with reduced activity still retains cytidine deaminase activity, resulting in some unintentional C-to-T mutations by TALEDs (Cho et al. 2022). On the other hand, mitoBE did not introduce C-to-T substitutions (Yi et al. 2023). As described later, a TALED approach was recently used to successfully edit target adenines in the plastid genome (Mok et al. 2022b).

## Targeted Editing of the Plastid Genome

More than 30 years ago, targeted disruption of plastid genes was achieved by a method called plastid transformation, in which foreign DNA fragments were inserted into the target site of the plastid genome [reviewed in (Maliga 2022)]. However, this method has been successfully used in only a few species and subspecies/ecotypes, and it is still difficult to use in monocots and model plants, including rice and *Arabidopsis* (Rascón-Cruz et al. 2021). Editing of the plastid genome by inserting a vector expressing the editing enzyme into the nucleus (**Fig. 1**) is probably applicable for more species than plastid transformation. A further benefit of employing genome-editing techniques for altering the plastid genome lies in the fact that certain countries do not categorize plants edited through this method as genetically modified organisms (GMOs), provided that the vector introduced into the nucleus can be subsequently removed.

A few studies have modified the plastid genome using TALENs. In one study, TALENs were used to improve the efficiency of plastid transformation by co-transforming genes of interest and a vector encoding TALENs into rice plastids (Li et al. 2016). Another group used plastid-targeted TALENs that were expressed from the tobacco nucleus to see whether TALENs are suitable for editing the plastid genome and to see repair processes after DSB occurred in the plastid genome (Huang et al. 2021). The DSB was repaired by homologous recombination (HR) including microhomology-mediated recombination, but not by non-homologous end-joining, which is predominantly observed in the nucleus. The following year, the same group reported analyses on the transcriptome of a heterotrophic albino mutant, in which *rpoB1* (a gene of a subunit of the plastid-encoded RNA polymerase) was knocked out by the plastid-targeted TALENs (Liu et al. 2022).

**Fig. 3:**
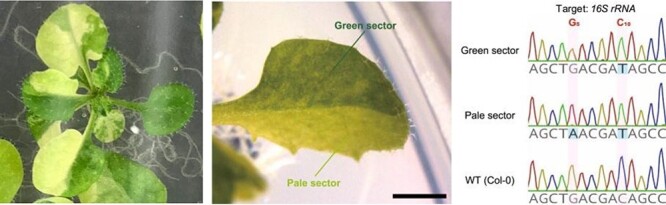
Base editing in the plastid genome sometimes causes chimeric phenotypes like leaf variegation. The pale sector in the middle panel has an additional G-to-A mutation as well as the C-to-T mutation observed in the green sector. Modified from Nakazato et al. (2021) with permission.

Two similar methods for targeted base editing in plastids were recently described in consecutive papers within the same issue (Kang et al. 2021, Nakazato et al. 2021), where both papers used DdCBE (Mok et al. 2020). In the first approach, Kang et al. (2021) succeeded in targeted C-to-T base editing in the plastid and mitochondrial DNA of protoplasts from rapeseed and lettuce in transient assays. A point mutation in *16S rRNA* to confer resistance to the antibiotic spectinomycin was introduced at an almost homoplasmic (99%) level in the plastid genome in regenerating shoots selected with spectinomycin. In the second approach, Nakazato et al. (2021) made a single Ti plasmid to express a pair of plastid-targeting DdCBE-like base editors [named plastid-targeted platinum TALE cytidine deaminases (ptpTALECDs)]. They then introduced the plasmid into the nucleus of *Arabidopsis thaliana* and obtained nuclear transformants, in which the target bases in the plastid genome were edited. In this case, the substitution in the T_1_ plants was found to be homoplasmic (**Fig. 3**), and off-target mutations in the plastid genome were low in number and frequency. In addition, all the tested T_2_ progeny inherited the point mutation at a homoplasmic level, and some did not have the nuclear-inserted base-editor gene. These T_2_ plants appeared to be so-called null segregants, which could be regarded as non-GMO genome-edited plants in some countries and thus would be beneficial for practical applications. Li et al. (2021) also used the plastid-targeting DdCBE to knock out the *psaA* gene in the plastid genome of rice regenerating shoots. Recently, Nakazato et al. (2023) reported that a modified version of ptpTALECD (named ptpTALECD_v2), in which an evolved version of DddA (Mok et al. 2022a) functioned as the cytidine deaminase domain, had higher base editing activity on both target and non-target cytosines than did ptpTALECD. Although editing of non-target cytosines is an undesired result, it was also suggested that ptpTALECD_v2 could generate plants in which only cytosines in the target window are edited. In addition, ptpTALECD_v2 could homoplasmically substitute GCs and CCs, some of which ptpTALECD could not do. Therefore, ptpTALECD_v2, like DdCBE and ptpTALECD, is another potentially valuable tool for modifying the plastid genome. Hu et al. (2023) reported another type of C-to-T base editor named cytidine deaminase–exonuclease–nickase–TALE (CyDENT). CyDENT could specifically edit cytosines on one strand of dsDNA, but its editing frequency in the plastid genome was much lower than the editing frequencies of DdCBE and ptpTALECDs [e.g. mutations were detected in up to 1.67% of next generation sequencing reads].

Another type of base editing, A-to-G editing, was achieved in the lettuce and *Arabidopsis* plastid genomes by using an Adenosine base editor named TALED, which was initially developed to edit mammalian mitochondrial DNA (Cho et al. 2022). A TALED consisting of two protein molecules was used to modify the plastid genome. One molecule comprised a TALE domain, the C-terminal half of DddA and a modified version of deoxyadenine deaminase (TadA-8e) originally derived from *Escherichia coli*. The other molecule comprised a TALE domain and the N-terminal half of the split DddA. DddA is thought to be necessary to unwind dsDNA so that TadA-8e can operate on it (Mok et al. 2022b). In this case, almost homoplasmic base editing was achieved in the T_1_ generation of *A. thaliana* and the next generation inherited the introduced mutations. Given that each cell contains more than 1,000 copies of the plastid genome (Zoschke et al. 2007), it is surprising that homoplasmic C-to-T and A-to-G base editing was achieved in just the first- or second-generation and that the mutations were stably inherited. These base editing methods could be regarded as effective and practical methods for modifying plastid genomes.

## Targeted Editing of the Plant Mitochondrial Genome

**Fig. 4:**
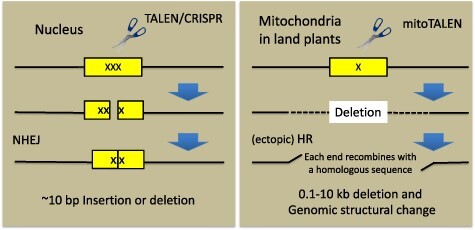
Comparison of the outputs after treatments of scissors-type genome editing between the nuclear genome (left) and the land plant mitochondrial genome (right). Abbreviation: NHEJ, non-homologous end-joining.

It is not yet possible to insert foreign genes directly into the mitochondrial genomes of multicellular organisms in a stable and inheritable manner. However, by employing a genome-editing method, mitochondria-targeted TALENs (mitoTALENs) were successfully used to precisely cut and eliminate disease-causing mutated mitochondrial DNAs in mammalian cell lines and in mice (Bacman et al. 2013, Reddy et al. 2015). At present, the most economically essential targets to be modified in the plant mitochondrial genome are genes responsible for CMS, which is a trait frequently selected for to produce F_1_ seeds. CMS lines are used as seed parents to prevent self or unwanted pollination efficiently and economically in crossing to the specific pollen lines in agriculture (Hanson and Bentolila 2004). CMS genes are also safe targets for editing because their disruption does not harm plant growth. The targets in the plant mitochondrial genome that were first edited by mitoTALENs were *open reading frame 79* (*orf79*) in BT-type CMS rice and *orf125* in rapeseed of kosena radish-type CMS, which have no sequence similarity but were shown to be responsible for CMS by reverse genetic genome-editing approaches (Kazama et al. 2019). When the two open reading frames were targeted by mitoTALENs, a large deletion hundreds to thousands of base pairs in size were detected in the target site, and in many plants, the remaining sequences did not reconnect with each other but recombined with distant sequences that were homologous to their end sequences (**Fig. 4**). These recombination events drastically changed the structure and gene order of the mitochondrial genome. Similar changes have not been detected after the cleavage of nuclear DNA and human mitochondrial DNA. Their occurrence in the plant mitochondrial genomes may be due to their having unique DNA repair processes and a higher frequency of (ectopic) HRs (Kubo and Newton 2008, Gualberto and Newton 2017).

Several mechanisms of CMS are known (Chen et al. 2017), and subsequent studies identified genes responsible for other types of CMS in rice and tomato by using mitoTALENs (Omukai et al. 2021, Kuwabara et al. 2022, Takatsuka et al. 2022). mitoTALENs has been used to edit genes other than CMS-responsible genes. For example, two genes for a ATP synthase 6, *atp6-1* and *atp6-2*, in *A. thaliana* ecotype Columbia-0 could be disrupted individually but not simultaneously, suggesting that these two genes are functionally redundant so that only one of them is essential for survival (Arimura et al. 2020). Ayabe et al. (2023) showed that knocking out the mitochondrial *nad7* gene of *A. thaliana*, which encodes a component of respiratory complex I, was lethal. In contrast, tobacco knockout mutants of mitochondrial *nad9* created by mitoTALENs lacked respiratory complex I, were non-lethal and showed CMS (Forner et al. 2023). Non-lethality and CMS were also exhibited by a *Nicotiana sylvestris* mutant lacking the mitochondrial *nad7* (CMS-II, Pineau et al. 2005). In tobacco, mitoTALENs in combination with chemical mutagens were successfully used to introduce several types of point mutations (C:G-to-T:A, C:G-to-A:T, T:A-to-C:G and T:A-to-A:T) in the TALE-recognizing sequence (Forner et al. 2022). In this study, the partial genome-edited lines, wherein mitoTALENs with limited activity persistently targeted mitochondrial DNA copies, yielded cleavage of only a restricted number of mitochondrial DNA copies. Subsequent chemical treatments induced stable and heritable point mutations within the TALE-recognition sites. This targeted random mutagenesis approach would be practical for investigating and enhancing specific genes or selective breeding purposes. Kang et al. (2021) first demonstrated that DdCBE could modify plant mitochondrial DNA. They transiently expressed mitochondria-targeting DdCBE in cultured cells of rapeseed and lettuce and introduced heteroplasmic mutations to the mitochondrial genome. Nakazato et al. (2022) introduced homoplasmic and inheritable mutations to the *Arabidopsis* mitochondrial genome by using a pair of mitochondria-targeting DdCBE-like base editors named mitochondria-targeted platinum TALE cytidine deaminase (mitoTALECD). In their paper, the authors substituted *atp1*-1178C with a T, which is known to be converted to uracil in the transcripts by a nuclear-encoded pentatricopeptide repeat protein, ORGANELLE TRANSCRIPT PROCESSING 87 (OTP87). The slow growth of *otp87* mutants, in which RNA editing at *atp1*-1178C is abolished (Hammani et al. 2011), was rescued by making a C-to-T substitution at *atp1*-1178C by mitoTALECD. In addition, RNA editing at *atp1*-1178C was inhibited by mutations introduced by mitoTALECD into the sequence that OTP87 was predicted to bind to (Takenaka et al. 2013). This result supports the working model for OTP87 in which OTP87 achieves RNA editing by binding to the sequence upstream of the RNA editing site. Although A-to-G base editing in the plant mitochondrial genome has not been reported yet, the base editors described earlier, including TALED and mitoBE, which work with mammalian mitochondrial DNA, should also work in plant mitochondria. The advantages are that they are also more precise and less disruptive than mitoTALENs.

## Conclusions and Perspective

The emergence of mitochondrial genome editing tools has at last enabled targeted stable modification of the mitochondrial genome of land plants, while the emergence of plastid genome editing tools has opened the door to modifying the plastid genome for species that have previously been recalcitrant to plastid transformation. Although the efficiency of genome editing by TALE-based genome editors can be high, assembling a TALE array is laborious and time-consuming. Thus, there is a need to develop a method for more easily assembling a TALE array or one to edit organellar genomes using the CRISPR/Cas system. New methods for modifying the mitochondrial and plastid genomes are also needed, other than cleavage and C-to-T and A-to-G base editing. Such strategies include foreign-gene insertion, replacement, introduction of a synthetic genome and search-and-replace genome editing like prime editing (Anzalone 2019).

Mitochondrial and plastid genomes are good targets for crop breeding. This is because they encode photosynthesis- and energy production–related genes and because modified organellar genomes are unlikely to escape via pollen due to the maternal inheritance in many species (Zhang et al. 2003). Genome editing tools can be of great value in crop breeding because some countries regard null segregants as non-GMOs. A good strategy for crop breeding utilizing organellar genomes would be to use sequential base editing to accumulate SNPs responsible for desirable agronomic traits. This has not yet been achieved because, in many species, all genes in both mitochondrial and plastid genomes are inherited uniparentally (mainly maternally) without any recombination. The rapid development of tools for modifying the underutilized mitochondrial and plastid genomes in recent years will lead to a better understanding of the functions of these genomes as well as improvements in crop breeding.

## Data Availability

No new datasets were generated or analyzed in this study.
